# Another Instance of Repudiation of the Nostrum Trade by an Apothecary

**Published:** 1848-11

**Authors:** 


*Another Instance of Repudiation of the Nostrum Trade by an Apothecary.*

We have the pleasure of announcing another instance in which an apothecary has renunciated all connection with the nostrum trade. Mr. A. J. Matthews, of this city, a highly respectable and competent druggist and appothecary, announces [see advertising sheet] that he has disposed of his entire stock of patent medicines, and will hereafter have nothing to do with them directly, or indirectly. These instances are gratifying as evincing the commencement of a reform, which will, in time, we have no doubt, be complete. Reflecting, conscientious dealers in drugs, have only to consider seriously the patent medicine system in its moral aspects, and they cannot feel that they have discharged their duty until they have washed their hands of all participation in the business. They who have taken the lead in this matter, with the risk, if not certainty of personal sacrifice, deserve great credit. If not rewarded by pecuniary profit, they will have the priceless approbation of their own consciences, and that respect which actions dictated by principle never fail to command.

